# Supplementary material for: Iridophore apoptosis mediates socially-regulated developmental color pattern plasticity in an anemonefish
Source: PLoS Biol. 2026 Feb 19;24(2):e3003630. doi: 10.1371/journal.pbio.3003630 (PMC12919797; doi:10.1371/journal.pbio.3003630)
Supplement: S1 Table — Letters denote environmental treatment, where “B” = ”Anemone”; “C” = “Fake/plastic anemone”; and “D” = “Empty”. (DOCX) [file pbio.3003630.s001.docx]

|  | Estimate | Std. Error | z value | Pr(>\|z\|) |
| --- | --- | --- | --- | --- |
| Intercept | -3.49 | 0.29 | -12.15 | <2e-16 |
| B | 1.50 | 0.29 | 5.24 | 1.60e-05 |
| C | 2.07 | 0.26 | 8.11 | 5.13e-16 |
| D | 2.17 | 0.28 | 7.88 | 3.75e-15 |
| sampled_age | -1.13 | 0.80 | -1.41 | 0.16 |
| sl_mm | -0.50 | 0.14 | -3.66 | 0.00025 |
| treatmentB:as.factor(sampled_age)62 | -1.32 | 0.94 | -1.41 | 0.16 |
| treatmentC:as.factor(sampled_age)62 | 1.37 | 0.79 | 1.74 | 0.082 |
| treatmentD:as.factor(sampled_age)62 | 1.25 | 0.80 | 1.56 | 0.119 |
