# Supplementary material for: Iridophore apoptosis mediates socially-regulated developmental color pattern plasticity in an anemonefish
Source: PLoS Biol. 2026 Feb 19;24(2):e3003630. doi: 10.1371/journal.pbio.3003630 (PMC12919797; doi:10.1371/journal.pbio.3003630)
Supplement: S2 Table — “LCL” = lower confidence limit; “UCL” = upper confidence limit. Letters denote environmental treatment, where “A” = “Occupied Anemone”; “B” = “Anemone”; “C” = “Fake/plastic anemone”; and “D” = “Empty”. (DOCX) [file pbio.3003630.s002.docx]

| Age | response | SE | LCL | UCL |
| --- | --- | --- | --- | --- |
| A:38dph | 0.03 | 0.008 | 0.017 | 0.051 |
| B:38dph | 0.12 | 0.018 | 0.089 | 0.16 |
| C:38dph | 0.19 | 0.031 | 0.14 | 0.26 |
| D:38dph | 0.21 | 0.034 | 0.15 | 0.29 |
| A:62dph | 0.01 | 0.007 | 0.002 | 0.042 |
| B:62dph | 0.01 | 0.009 | 0.003 | 0.052 |
| C:62dph | 0.24 | 0.036 | 0.17 | 0.31 |
| D:62dph | 0.23 | 0.04 | 0.17 | 0.31 |
