# Supplementary material for: Iridophore apoptosis mediates socially-regulated developmental color pattern plasticity in an anemonefish
Source: PLoS Biol. 2026 Feb 19;24(2):e3003630. doi: 10.1371/journal.pbio.3003630 (PMC12919797; doi:10.1371/journal.pbio.3003630)
Supplement: S3 Table — Data excludes the outlier individual (C7). (DOCX) [file pbio.3003630.s003.docx]

|  | Estimate | Std. error | z value | Pr(>\|z\|) |
| --- | --- | --- | --- | --- |
| (intercept) | -0.71 | 0.14 | -5.00 | 5.68e-07 |
| Treatmenthigh dose | -0.58 | 0.21 | -2.78 | 0.0054 |
| Treatmentlow dose | -0.024 | 0.24 | -0.10 | 0.9211 |

replicate: variance = 3.69e-16, Std. Dev. = 1.92e-08.
