# Supplementary material for: Iridophore apoptosis mediates socially-regulated developmental color pattern plasticity in an anemonefish
Source: PLoS Biol. 2026 Feb 19;24(2):e3003630. doi: 10.1371/journal.pbio.3003630 (PMC12919797; doi:10.1371/journal.pbio.3003630)
Supplement: S4 Table — (DOCX) [file pbio.3003630.s004.docx]

|  | Estimate | Std. error | z value | Pr(>\|z\|) |
| --- | --- | --- | --- | --- |
| (intercept) | 1.06 | 0.14 | 7.54 | 4.83e-14*** |
| bar_no | -2.04 | 0.19 | -10.78 | <2e-16*** |
| delta_SL | 0.17 | 0.11 | 1.60 | 0.11 |

trial_ID: variance = 0.095, Std. Dev. = 0.31; tank_ID: variance = 1.16e-10, Std. Dev. = 1.08e-05.
