# Supplementary material for: Iridophore apoptosis mediates socially-regulated developmental color pattern plasticity in an anemonefish
Source: PLoS Biol. 2026 Feb 19;24(2):e3003630. doi: 10.1371/journal.pbio.3003630 (PMC12919797; doi:10.1371/journal.pbio.3003630)
Supplement: S5 Table — (DOCX) [file pbio.3003630.s005.docx]

|  | Estimate | Std. error | z value | Pr(>\|z\|) |
| --- | --- | --- | --- | --- |
| (intercept) | -1.48 | 0.32 | -4.63 | 3.69e-06*** |
| delta_SL | 0.33 | 0.17 | 1.91 | 0.06 |
| bar_no | 2.56 | 0.31 | 8.39 | <2e-16*** |

trial_ID: variance = 5.21e-10, Std. Dev. = 2.28e-05; tank_ID: variance = 0.18, Std. Dev. = 0.42.
