# Supplementary figures and images for: Iridophore apoptosis mediates socially-regulated developmental color pattern plasticity in an anemonefish
Source: PLoS Biol. 2026 Feb 19;24(2):e3003630. doi: 10.1371/journal.pbio.3003630 (PMC12919797; doi:10.1371/journal.pbio.3003630)

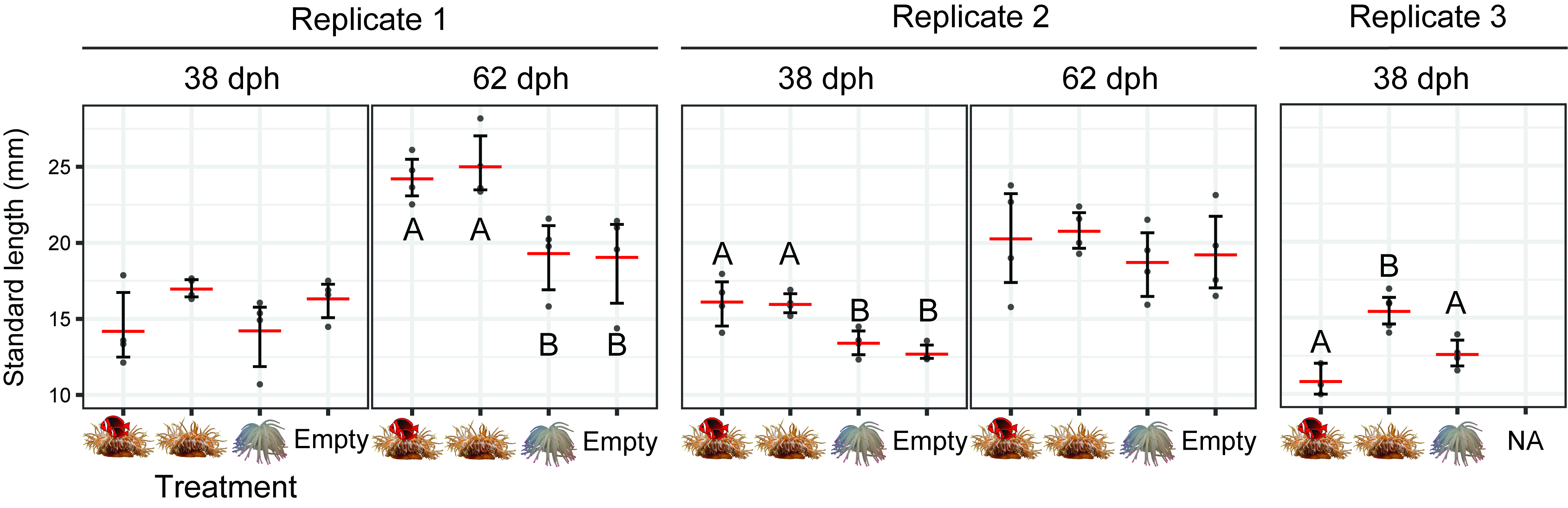

Supplement: S1 Fig — Juvenile standard length per environmental treatment per replicate at 38 and 62 days-post-hatch/dph. Letters denote statistical significance (ANOVA, padj < 0.05) grouping. The data underlying this Figure can be found in https://doi.org/10.5281/zenodo.17973175. (TIF) [file pbio.3003630.s006.tif]

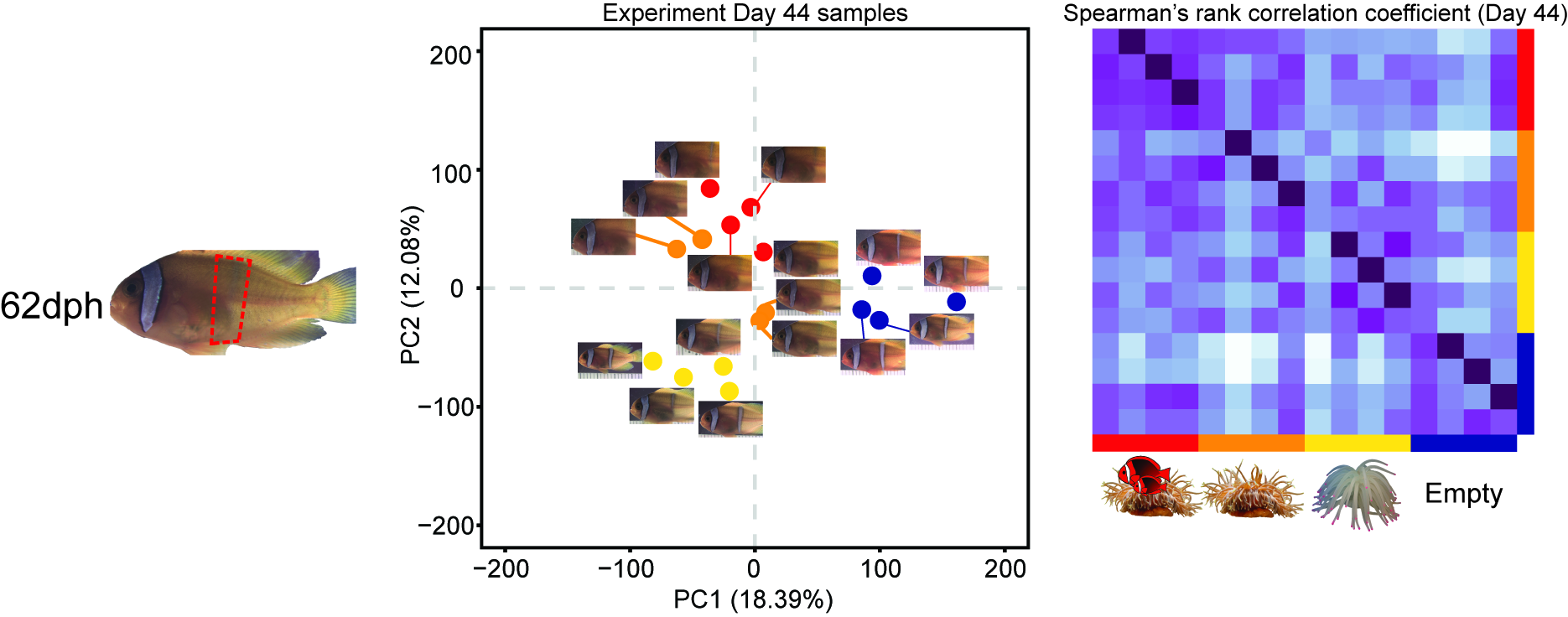

Supplement: S2 Fig — PCA showing the distribution of Day 44 (62 dph) body bar skin samples along PC1 and PC2 according to total gene expression, along with correlation matrix depicting pairwise Spearman’s rank correlation coefficients, calculated using transcriptomic data. The data underlying this Figure can be found in https://doi.org/10.5281/zenodo.17973175. (TIF) [file pbio.3003630.s007.tif]

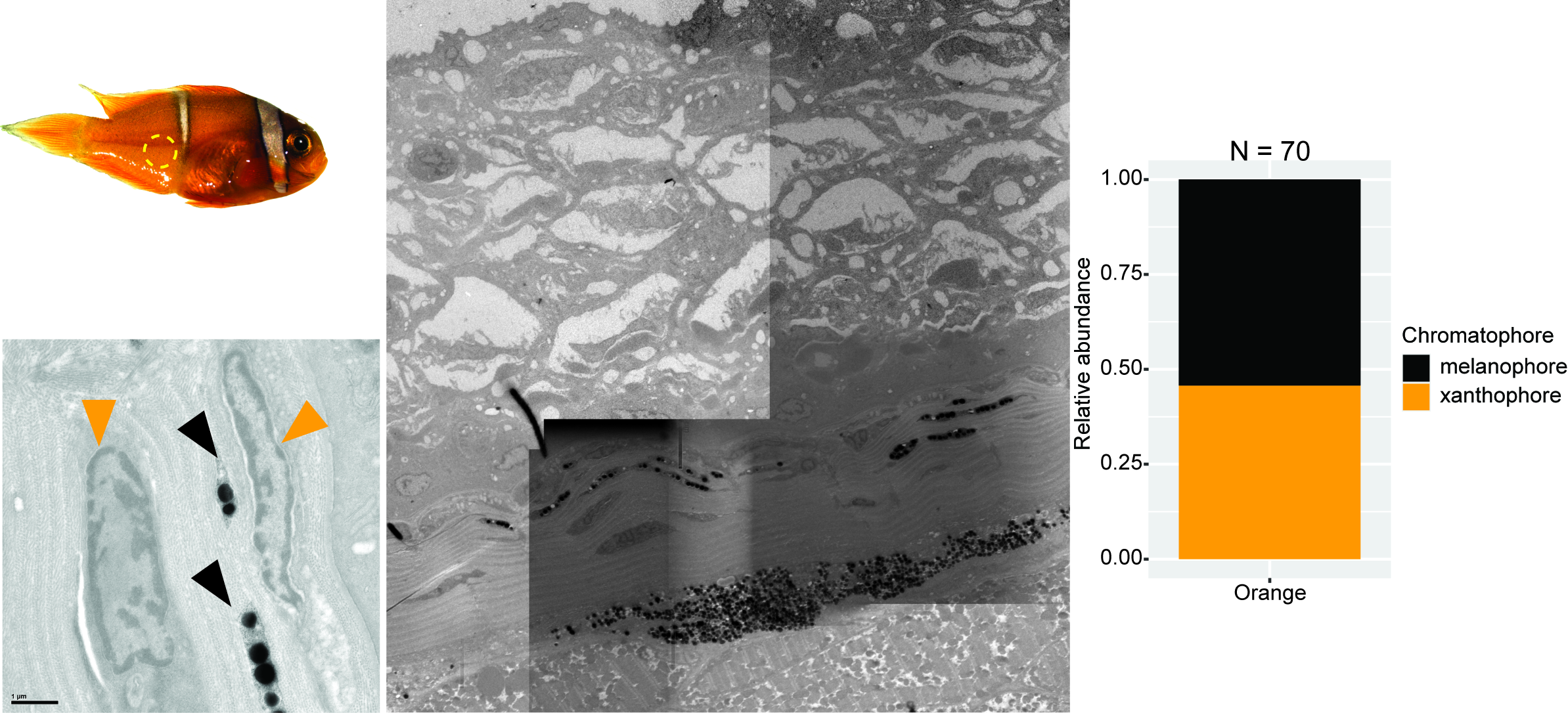

Supplement: S3 Fig — Example of a TEM section in the orange skin, peripheral to the fading white bar, with a close-up showing pigment cells (triangles). No iridophores were detected. Bar plot shows the proportional abundance (counts) of different chromatophore types counted from 70 cells in three sections. The data underlying this Figure can be found in https://doi.org/10.5281/zenodo.17973175. (TIF) [file pbio.3003630.s008.tif]

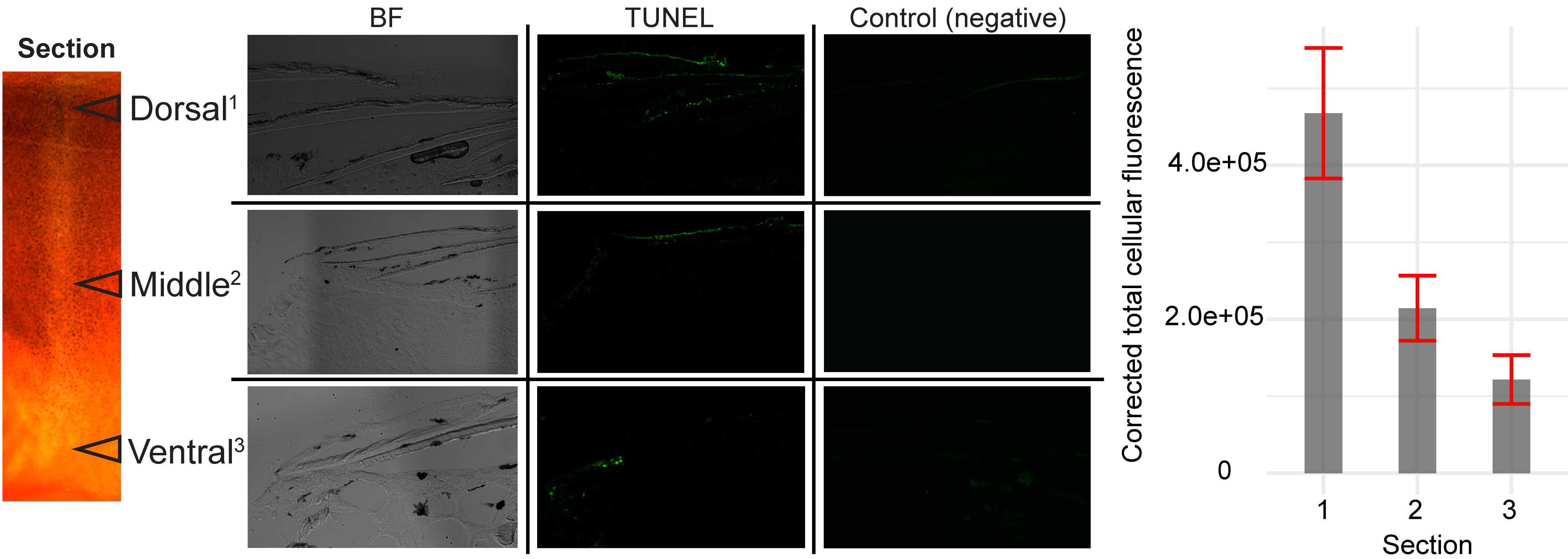

Supplement: S4 Fig — Three Sections (10 μm thick) were made in dorsoventral regions of the fading body bar of A. frenatus, including brightfield “BF,” TUNEL reaction mix-treated, and negative control. The side bar plot depicts the mean (±0.95 CI) area corrected total cellular fluorescence (CTCF) measured from individual cells across three sections per the ventral (section 1, n = 64), middle (section 2, n = 75), and dorsal (section 3, n = 63) regions. The data underlying this Figure can be found in https://doi.org/10.5281/zenodo.17973175. (TIF) [file pbio.3003630.s009.tif]

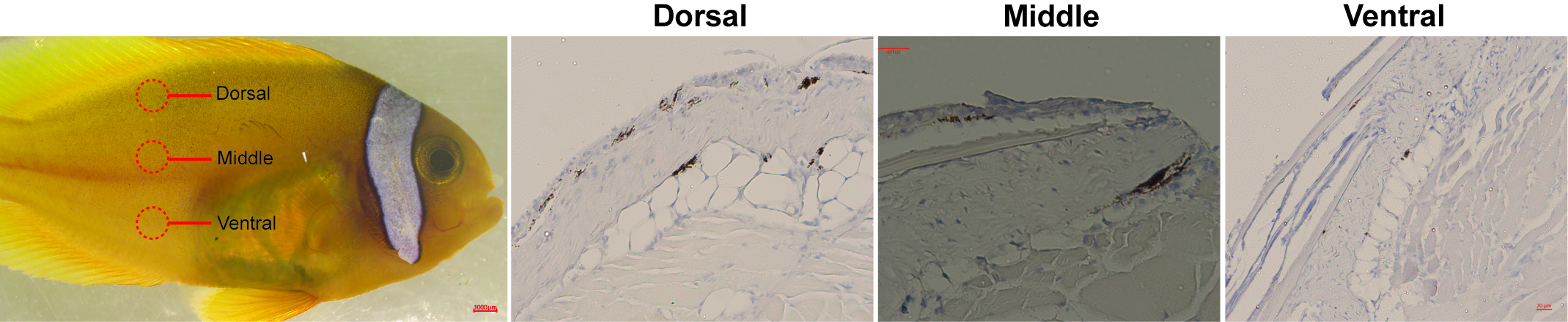

Supplement: S5 Fig — No DAB+ cells were observed. The data underlying this Figure can be found in https://doi.org/10.5281/zenodo.17973175. (TIF) [file pbio.3003630.s010.tif]

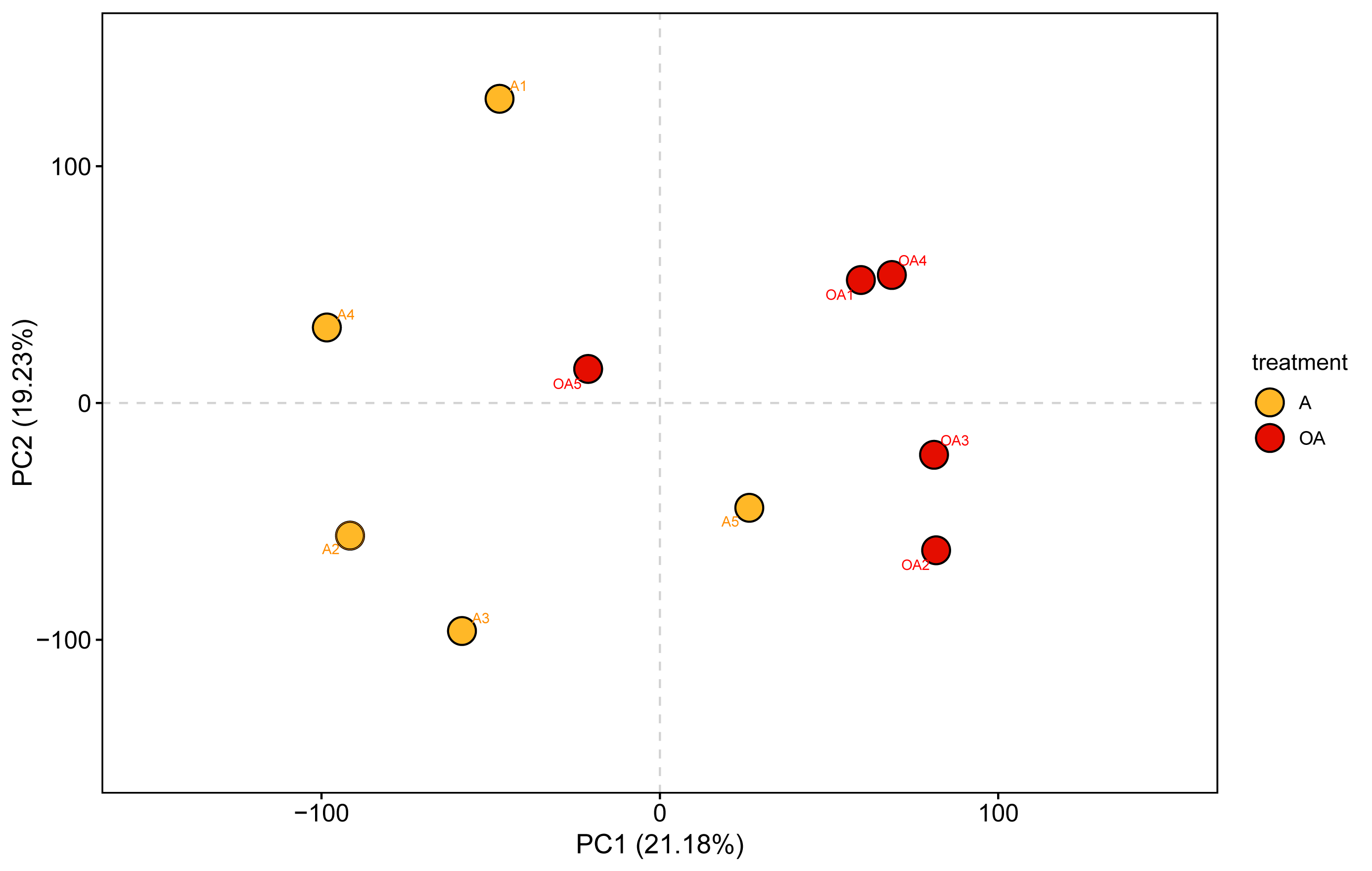

Supplement: S6 Fig — The data underlying this Figure can be found in https://doi.org/10.5281/zenodo.17973175. (TIF) [file pbio.3003630.s011.tif]

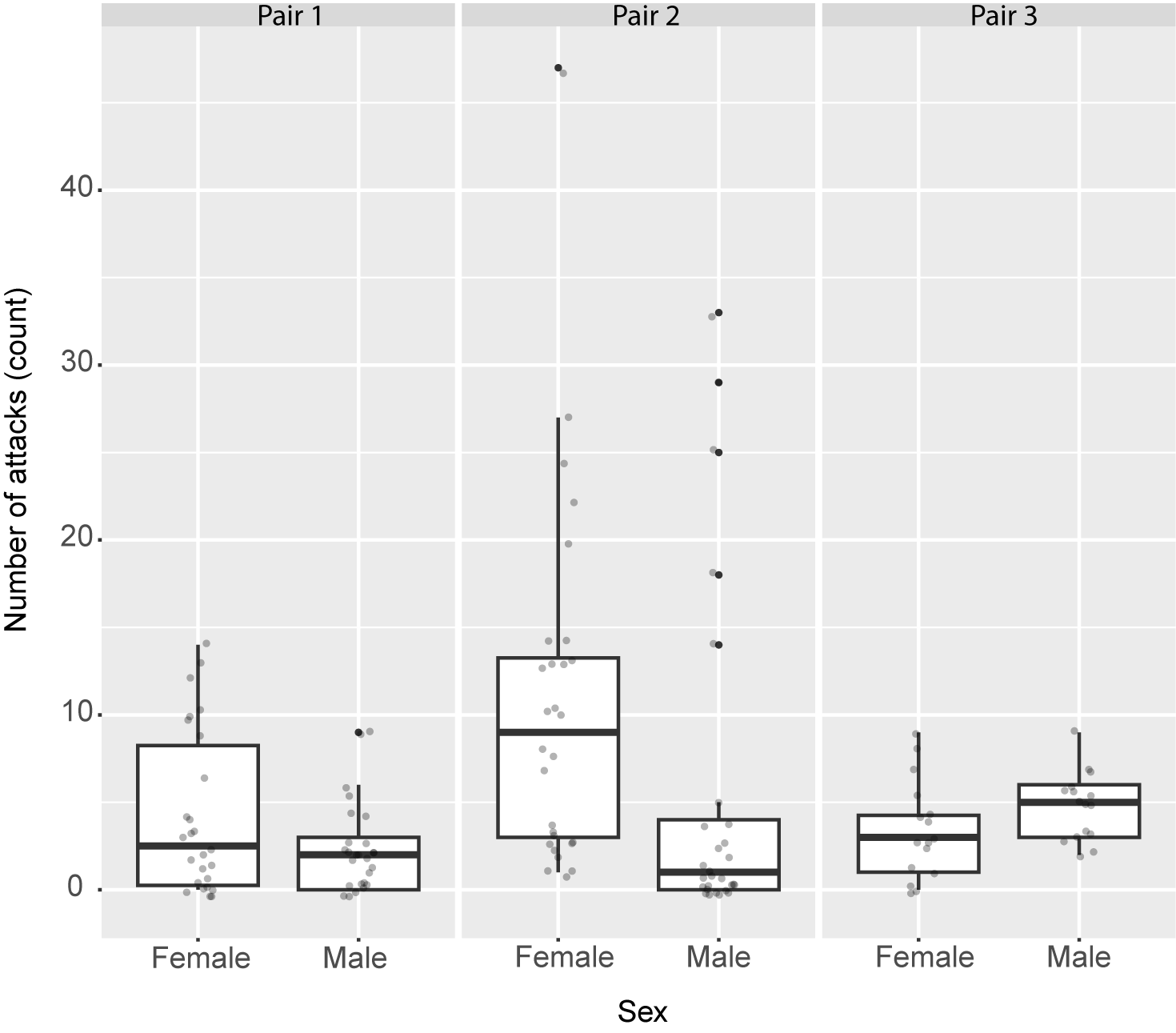

Supplement: S7 Fig — Presented is the combined behavior data from Experiments 1 and 2. Boxes represent the median, 25th and 75th percentiles, and range (whiskers). Points represent the summed number of attacks per trial. The data underlying this Figure can be found in https://doi.org/10.5281/zenodo.17973175. (TIF) [file pbio.3003630.s012.tif]

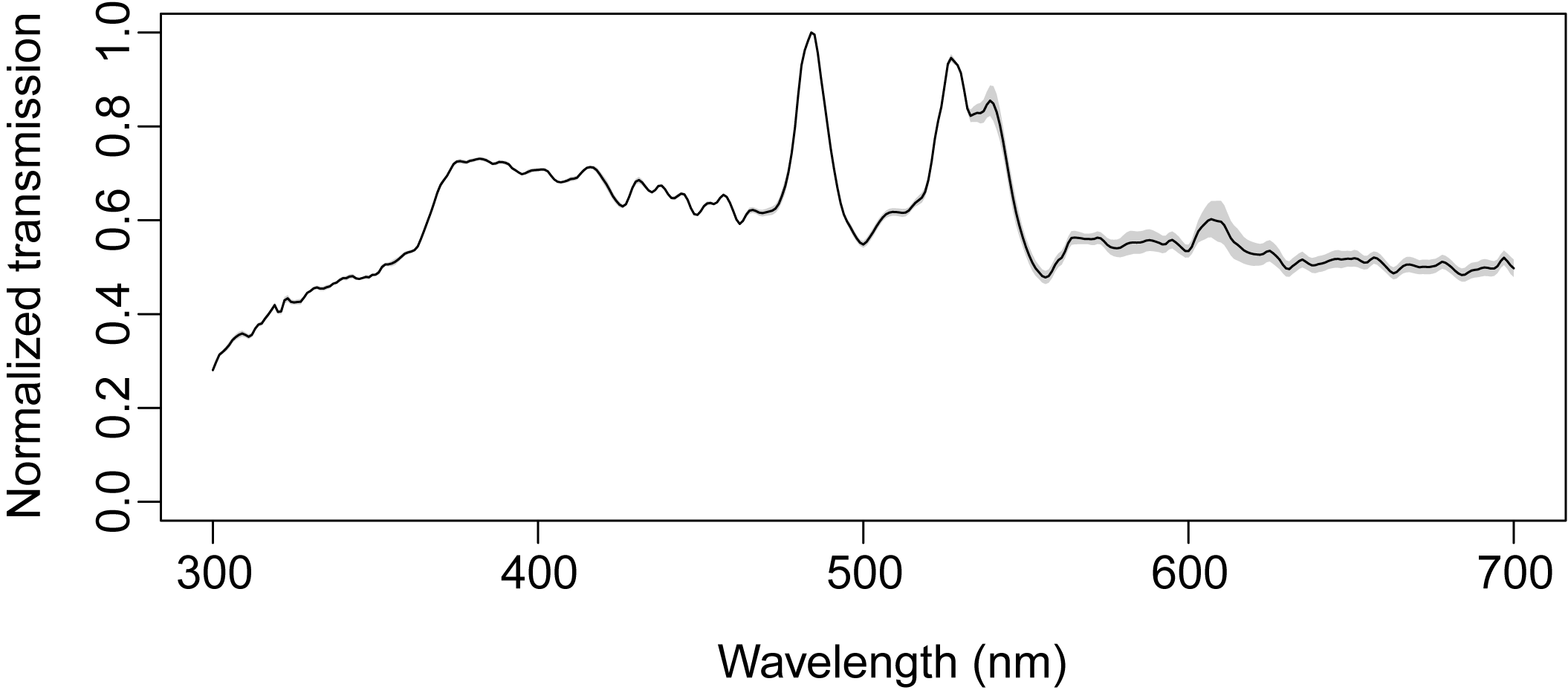

Supplement: S8 Fig — Shown is the average transmission spectra (n = 3) with standard error shaded in gray. The data underlying this Figure can be found in https://doi.org/10.5281/zenodo.17973175. (TIF) [file pbio.3003630.s013.tif]

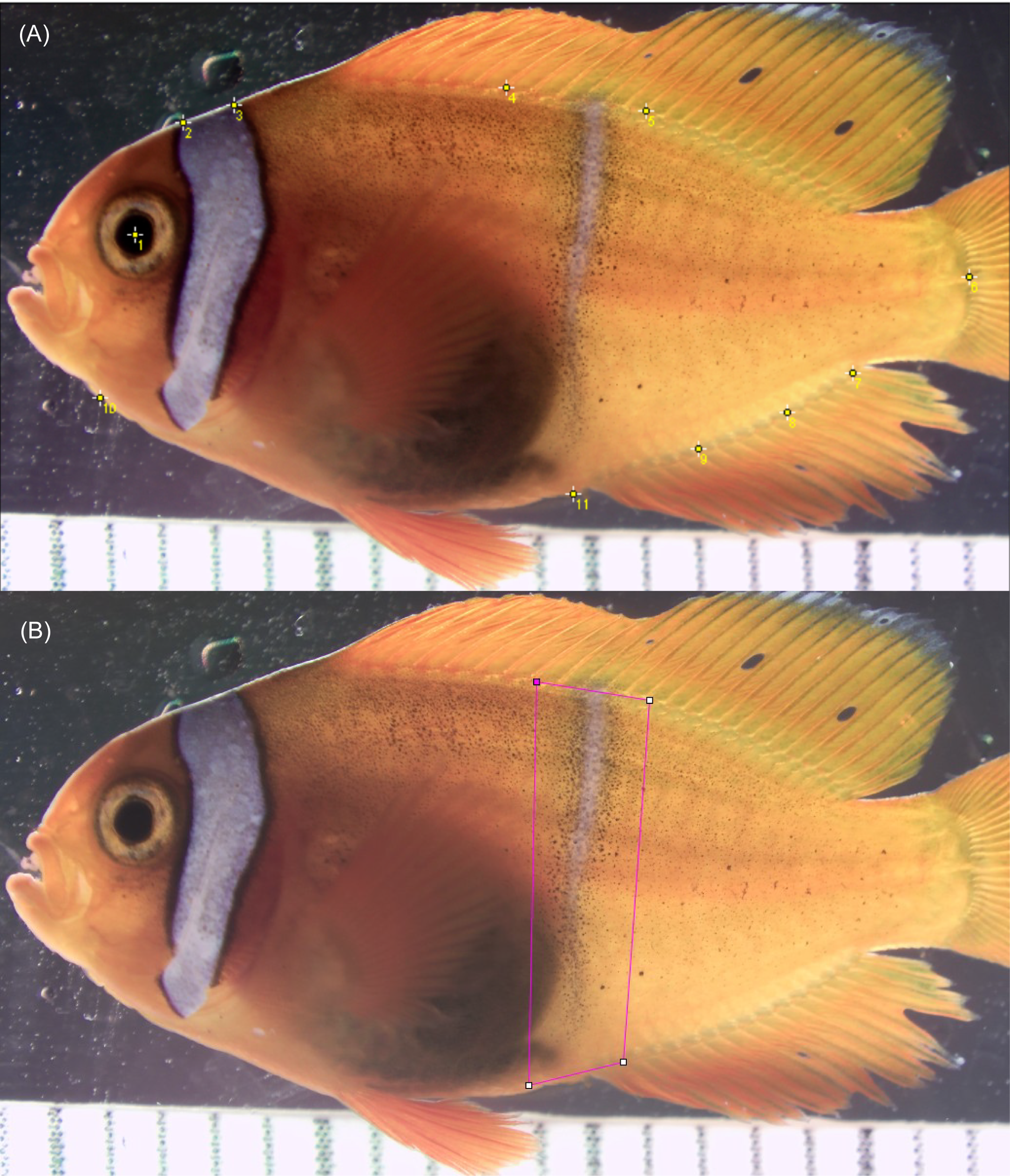

Supplement: S9 Fig — (A) Landmarks used for the alignment of fish images in color patch area quantification. (B) Vector locations for the mask used for isolating the body bar region were dorsally at the 8th dorsal spine and 4th dorsal ray, and in-line ventrally with the 2nd anal fin spine and posterior-edge of the pelvic fin. The data underlying this Figure can be found in https://doi.org/10.5281/zenodo.17973175. (TIF) [file pbio.3003630.s014.tif]
