# Supplementary material for: Iridophore apoptosis mediates socially-regulated developmental color pattern plasticity in an anemonefish
Source: PLoS Biol. 2026 Feb 19;24(2):e3003630. doi: 10.1371/journal.pbio.3003630 (PMC12919797; doi:10.1371/journal.pbio.3003630)
Supplement: Attachment — Submitted filename: Address_to_reviewer_comments_auresp_3.docx [file pbio.3003630.s018.docx]

**Address to reviewer comments**

**Reviewer #1: PBIOLOGY-D-25-02402R1**

In this interesting, comprehensive, well-written and well-presented manuscript, Mitchell and colleagues use an integrative approach to investigate how social environment influences a key developmental trait: the loss of a white bar during the juvenile stage in an anemonefish. Juveniles living with adults lost their bar earlier than those kept in isolation, suggesting this is a socially-mediated form of phenotypic plasticity.

The researchers use a strong integrative approach to explore the mechanisms behind this change spanning transcriptomic analyses, transmission electron microscopy and pharmacological inhibition, that all pointed to the massive apoptosis of iridophores as a key mechanism in this color transition. Finally, he authors also explored the ecological and evolutionary implications of this plastic trait. Behavioral trials showed that adult anemonefish respond differently to juveniles with one versus two bars, but paradoxically adults showed heightened aggression to those that exhibited bar loss. They also used phylogenetic approaches to look at the evolution oof this traits and found that it is strongly associated with clownfish that form smaller social groups. The authors suggest that perhaps this developmentally plastic trait has some function during recruitment, perhaps mitigating conflict between recruit-stage juveniles and adults where there are fewer juveniles and thus less social buffering.
Overall, I was impressed by the extent and quality of the work, particularly the multifaceted work undertaken to explore mechanism. The molecular work is very solid and compelling - I really like the combination of transcriptomics, TM and pharmacological intervention to explore mechanism and I have no suggestions for improvement there.

*We would like to thank the Reviewer for giving their time and effort to appraise our manuscript. The comments given are insightful and have even offered helpful directions to expand on our study and improve the overall quality of our revised manuscript.*

*Below in the highlighted sections, we have given a comment-by-comment address to each of the reviewer’s suggestions/concerns, and in quotes given the revised sections.*

However, there are a few things in the behavioral experimentation and conclusions that I was less convinced about or found unclear.

First, the authors do not make it particularly clear what the rationale is for the What is the rationale for the different environmental treatments. Presumably anemone and fake anenome are used to test for role of host on the color change, but I could not see this explicitly stated. I think it would be good to clarify this in revision.

*The reviewer’s intuition here is correct. Our four treatment groups: occupied anemone (with adults), unoccupied anemone (without adults), plastic/fake anemone, and anemone altogether tested different aspects of the environment on the plasticity in timing of bar loss. Our two live anemone treatments were to tease apart the influence of the host versus top-down social dominance (from adults), the fake anemone to identify the effect of a live host (symbiosis), and empty tank to isolate the effect of habitat/shelter.*

*We have since added two sentences (see Lines 142-147) near the beginning of the results section to clarify the reasoning for including the four treatments*:

***“Treatments 1 and 2 recreated the natural settlement conditions experienced by juveniles returning to the reef, where they settle into either an inhabited or uninhabited host. This enabled isolating the effect of top-down social pressure on bar loss by adult presence/absence in natural habitat. In addition, the inclusion of treatments 3 and 4, isolated the importance of a live host (symbiosis) from the availability of structural habitat (shelter).”***

Second, given the hypothesised importance of social environment to this color change process I was surprised that the "occupied" treatment was not replicated across the different settings. I think this is a significant weakness of that part of this study and it should be acknowledged by the authors in the results and discussion. To be clear, I am not advocating that the work be repeated with those additional treatments - that would be way too onerous, but the limitation should be dealt with explicitly in revision.

*Ideally, we would have included each of the fake anemone and empty treatments with adult occupancy. However, these were initially considered but were ultimately dropped for multiple reasons relating to both ecological relevance and practical issues. In particular, larval survival and production issues relating to our broodstock at the hatchery facility meant that we lacked enough additional (naïve) juveniles to support further treatment groups. As such we prioritised the use of our juveniles in the two naturalistic conditions (with/without adults and a live host), and two unnatural conditions to help separate the influence of a live host versus structural habitat/shelter. The decision to drop the non-anemone conditions (with adults) was also made to limit the number of highly artificial treatments in our experiment that had no ecological relevance (as anemonefish are obligate symbionts on the reef). Considering the above, we also thought it an unnecessary high-risk to the welfare of the animals, as our observations and those from published studies has found that with poorer quality or smaller habitat that mature anemonefish become less tolerant of juveniles and the likelihood of (violent) rejection increases, especially within the confines of an aquarium. We have now acknowledged this as a limitation in our study (see Lines 752-762):*

***“One limitation in our experimental design was the lack of replicating the adult occupancy without a live host anemone. Thus, we could not fully separate the interacting effects of a host and social conditions on bar loss. Future work isolating the effects of sociality from symbiosis could aid in fully characterizing the mechanistic basis of the developmental plasticity. However, this comparison would provide little-to-no ecologically relevant insights into the plasticity of bar loss, as all anemonefishes are obligatory symbionts meaning that the local host and social conditions are inextricably linked. Additionally, small and/or poor-quality hosts can limit group size and recruitment [82,83], as dominant anemonefish increasingly forcibly evict subordinates or reject recruits [84]. The heightened risk of adults in artificial habitat attacking juveniles raises practical and ethical concerns.”***

Realted to the above on line 205 the authors state "…our results suggest that the juvenile fish in response to perceiving their socially enriched environment accelerated their transition from the two-bar to the one-bar phenotype." This seems to be mostly supported by loss of bar stripes in the one social tank versus the others. Likely this finding  is true, but replication of the occupied social state would strengthen. I note each tank setup was replicated n=2 times, but n=3 would likely have provided a stronger statistical framing.”

*Again, we fully agree with the reviewer on both points, and regarding the lack of replication under the occupied social condition we refer to our detailed response above. The same practical constraints previously affected our ability to perform a third replicate. This situation is due to two concurrent and unexpected factors. First, our husbandry facility has recently faced an unforeseen shortage of personnel, which has reduced our capacity to maintain optimal larval-rearing conditions. Second, winter has arrived exceptionally early this year in Okinawa, and it is well known in our system that both spawning quality and larval survival decline sharply when seasonal conditions change abruptly. However, since our initial submission, we were able to rear a single very small clutch and conduct a third replicate for the development experiment. However, because there were so few larvae reared to the juvenile stage, we decided to drop one treatment group to ensure enough juveniles were allocated. In this case, we dropped the Empty treatment, as it previously showed virtually no difference in the rate of bar loss to the Fake anemone treatment. Unfortunately, high mortality during this third replicate meant that we only had enough juveniles to sample at the 38 dph stage and had to terminate the experiment before reaching 62 dph. However, the differences in bar loss were consistent with the previous replicates. We have since re-run related statistical analyses and updated statistical results to account for this third replicate. We also added mention of the truncated third replicate in the figure legend see Lines 165-166:*

***“The third replicate was conducted till 38 dph and excluded the Empty treatment.”***

*In the results, see Lines 186-187:*

***“Due to high mortality of juveniles in the third replicate (n = 3-5 survivors), it was limited to 20 days and had to drop one treatment (Empty).”***

*and in the methods, see Lines 921-923:*

***“Because the third replicate had a very low larval rearing survival, it was decided to limit the experiment to three treatments and exclude the Empty treatment.”***

*and Lines 927-929:*

***“However, the third replicate was terminated on Day 20 due to multiple mortalities in all three treatments (Occupied anemone, n = 5; Anemone, n = 3; Fake anemone, n = 5).”***

*In principle, we would be willing to conduct an additional (fourth) complete replicate. However, over the past months we have experienced major difficulties with our larval rearing (as outlined above), which prevented us from producing a normal sized clutch. While these problems are being addressed, we are also currently out of season and breeding pairs have largely halted spawning activity that will unlikely resume till May (2026). Consequently, we are limited in our ability to raise additional larvae to run another (full-sized) replicate and other experiments.*

*In addition, we must remind the reviewer that this experiment is extremely time-consuming: each replicate requires approximately two months to complete, or nearly three months when including the larval rearing period. Owing to limited allocated space, replicates cannot be run in parallel, meaning that the two initial replicates required close to six months (and ~1-month for Replicate 3). Taking these constraints into account and given the clear and consistent differences already observed in bar-loss rates, we believe that another replicate would unlikely alter the main results and conclusion.*

*Nevertheless, if the reviewer and/or editor considers an additional full replicate is essential, we are willing to attempt it, provided that an extension of at least 4-months can be granted.*

I was puzzled by the experiment illustrated in figure 6, that shows, perhaps counter-intuitively, that juveniles that had transitioned to one bar were more likely to be attacked than the two barred individuals. The authors do not explore this as much as I thought they might, stating that this developmentally plastic trait might be linked to recruitment. I suspect this is probably on track, but is it not more likely that the two-bar morph is a sign of the juvenile stage and one-bar of the adult form? If it is, might you have observed more aggression in the occupied tank, and might the aggression, mediated via the stress axis, be a mechanism though which bar loss is accelerated? One way to tease this apart would be conduct experiments where individuals can see each other but not interact, to determine if it is aggressive interactions or just the visible presence of adult fish that accelerates the color change. I also wondered how you controlled for size and sex in these experiments?

*Likewise, we were also very surprised by this result. One interpretation is that it fulfils a very short-lived function only during the recruitment phase for juveniles when they arrive at the anemone and the coming weeks to facilitate social integration into the pre-existing group. Alternatively, lone settlers may benefit from delaying the loss of supplemental barring as a safe strategy to maximise growth while minimising harassment and the risk of being displaced by roaming adult(s). We cover these ideas in the discussion (Lines 785-797). Certainly, there are many further areas to investigate in the future regarding this system and how it functions on the reef.*

*We agree that the one bar stage resembles the adult form. From the adult fish perspective, the one bar juveniles likely appear as being closer to adult-like, and more-recognisable as a conspecific leading to the higher aggressive response. Hirose (1995) study similarly postulated the idea that the colour pattern change in A. frenatus is related to rank ascension. These ideas are complementary to the social camouflage hypothesis originally proposed by Hans Fricke (1980) in emperor angelfish, and since thought to be employed by multiple other species of territorial reef fishes (see Lines 773-777). We have since added a brief sentence to synthesize the idea of appearing distinct from adults with importance in recruitment an (see Lines 777-779):*

***“…Similarly, the multiple white bars of A. frenatus juveniles may confer a recruitment advantage by appearing distinct from the adults, while the one bar morph is more recognizable as a conspecific.”***

*We tried comparing levels of overall aggression between our developmental treatments from in-tank video footage. However, we did not find any meaningful differences in the frequencies of aggressive interactions (chases, biting) among our treatments. However, this seemed to be an issue of an often-obstructed view of juveniles caused by live anemones that made it difficult to conduct a fair comparison among the treatments. Nevertheless, it was frequently observed that adults harassed/chased juveniles (see Lines 148-149; Lines 727-728), a type of interaction exclusive to the occupied anemone treatment.*

*For the reasons outlined above—namely the unexpected difficulties in larval production, the early seasonal cessation of spawning in Okinawa, and the time-consuming nature of these experiments—we were unfortunately not able to conduct the additional experiment suggested. However, we fully acknowledge its potential value and will consider incorporating such an approach in future studies to identify the involved social cue when conditions allow. We have added a sentence suggesting that future investigation should aim at isolating the specific social cue driving the response (see Lines 734-737):*

***“Future investigation into the role of stress in bar loss should compare the effect on juveniles when exposed to isolated adult visual cues (i.e., able to see but not interact with adults) to reveal whether physical harassment triggers accelerated bar loss.”***

*Although we considered that the stress axis driven by adult aggression could be a mechanism accelerating bar loss (see Lines 732-734), there were no clear differences in major corticosteroid-related gene expression levels at the whole-body level when comparing juveniles that shared habitat with/without adults (see Lines 533-536).*

*As anemonefishes are protandrous hermaphrodites (male to female), there was no need to control for the sex of the juvenile subjects. The juveniles used in our experiments would have been in an ambisexual state as they had not enough time for the gonads to mature and transition into males (this typically takes between 6-12 months without social suppression). Juveniles used in the developmental experiment were 18 days-post-hatching at the beginning of each replicate. Those used in the behaviour experiment were between 2-5 months of age and were kept in socially suppressive conditions with numerous (~30 individuals) in a single tank that prolongs the juvenile stage by preventing pair and territory formation.*

*Adult fish used in the developmental experiment were stable pairs that had established a clear size difference (3-4cm) between the female and male. While the pairs had not exhibited prior spawning behaviour, they displayed typical courtship behaviours of breeding pairs. Adult pairs used in the behaviour experiment were active broodstock fish with prior spawning history. Note that adult fish were excluded in behavioural trials if they had a clutch of eggs. Adults and older juvenile fish were measured in standard length by photographing them individually in a thin plastic tank that had a printed-on scale (in mm) (see Lines 1111-1113).*

*The size of the juveniles in the developmental experiment could not be accurately measured (under a microscope) due to their age (18dph) and small size (<10mm) that meant they were extremely fragile and at risk of excess mortality from fright (they are very prone to heart attacks from even careful movement at this age). However, we did make approximate measurements of juveniles in each treatment cohort by photographing them overhead in a shallow tray containing a ruler prior to acclimation into the experimental tanks. The mean size differences between treatments in both replicates were within <1mm. We have now included details of the mean (±SD) total lengths of each treatment cohort in the methods (see Lines 903-913):*

***“******Because of the fragility of juveniles and high risk of mortality, it was not possible to accurately measure the initial body lengths of fish assigned to each treatment using a microscope. Approximate measurements were instead taken from overhead photographs using a phone camera of the juveniles in a shallow dish containing a tape measure. There were small (<1mm on average) differences among the initial standard lengths of juveniles in Replicate 1 (SL mean ± sd, Occupied anemone = 8.27 ± 0.45 mm; Anemone = 8.29 ± 0.52 mm; Fake anemone = 8.05 ± 0.67 mm; Empty = 7.92 ± 0.66 mm), Replicate 2 (Occupied anemone = 8.17 ± 0.61 mm; Anemone = 8.13 ± 0.65 mm; Fake anemone = 8.07 ± 0.68 mm; Empty = 8.15 ± 0.84 mm), and Replicate 3 (Occupied anemone = 9.17 ± 0.71 mm; Anemone = 8.96 ± 0.63 mm; Fake anemone = 8.99 ± 0.67 mm).”***

*In addition, we took more accurate (microscope-taken) measurements of the baseline (pre-treatment) fish (n = 4) (see lines 914-918). These (18dph) pre-treatment fish were randomly sampled (for RNA extraction in Replicate 1) immediately prior to initiating the experiment and indicated that body sizes were reasonably uniform within each cohort.*

***“More accurate (microscope-taken) measurements of four randomly sampled (for RNA sequencing) pre-treatment juveniles indicated there was reasonable uniformity among initial body lengths (Replicate 1 = 8.70 ± 0.15 mm; Replicate 2 = 7.85 ± 0.26 mm; Replicate 3 = 10.39 ± 0.56 mm).”***

Reviewer #2 [Sebastian Gaston Alvarado]: This is an ambitious and well-executed study that integrates molecular, cellular, behavioral, and phylogenetic approaches to demonstrate that ontogenetic bar loss in the tomato anemonefish is a socially regulated, plastic trait. The work convincingly establishes that the apoptosis of iridophores, mediated in part by thyroid hormone signaling, is the proximate mechanism for bar loss, while behavioral trials and evolutionary analyses highlight the ecological and evolutionary relevance of transient barring. The integration of these diverse datasets provides a compelling eco-evo-devo case study with broad significance for understanding plasticity in animal coloration.

That said, I have noted a handful of minor comments that can be addressed through clarifications in the text:

*We thank the reviewer for giving their time to appraise our manuscript and appreciate their kind words. Below we address each of their comments and highlight areas where the revised manuscript has been improved/edited accordingly.*

Line 586-596 (Comparative phylogenetics): "…revealed that species with bar loss during ontogeny have larger mean group sizes…"
Comment: The wording currently suggests causation. The authors should reframe this as a correlative association and acknowledge alternative ecological factors that could also influence group size and color pattern evolution.

*Good point, we should be more careful with our wording to avoid attributing causation. We have modified this sentence to not infer causation (see Lines 612-613):*

**“… species with bar loss during ontogeny are associated with having larger mean group sizes”**

*We agree with the Reviewer, colour and colour patterns are highly complex traits both in form and function. Rarely (if ever) do they have a single function, and this almost certainly holds true with anemonefishes. We have now added mention in the discussion that host size is known to limit group size (see Lines 760-762):*

***“…small and/or poor-quality hosts can limit group size and recruitment [82,83], as dominant anemonefish increasingly forcibly evict subordinates or reject recruits [84].”***

*And we added mention that specific host anemone traits might have contributed to the evolution of bar loss during ontogeny (see Lines 802-806):*

***“While we did not find evidence of bar loss during ontogeny being linked to host specificity or the number of host associations, it remains to be examined whether preferred host traits (e.g., area, coloration, toxicity, tentacle length) contributed to its evolution, as has been suggested for other anemonefish color pattern traits (see [35,30]).”***

Line 605(Discussion): "Here, we revealed environmentally induced temporal plasticity in a convergent, juvenile-to-adult reef fish color pattern transition."
Comment: This phrasing generalizes beyond the focal species. The authors should qualify that the mechanism was demonstrated specifically in Amphiprion frenatus, while suggesting that similar processes could occur across anemonefishes.

*We have now revised this sentence to specify that the mechanism was demonstrated in A. frenatus (see Lines 628-630):*

***“Here, we revealed environmentally induced temporal plasticity in the tomato anemonefish (A. frenatus) that exhibits a convergent, juvenile-to-adult reef fish color pattern transition.”***

Line 610-611: "…increased TH production related gene expression and activity in the skin might form a neuroendocrinal link between the perception of social cues and shifts in chromatophore populations."
Comment: This implies direct hormone measurement, but the evidence is gene expression only. The authors should clarify that their inference of endocrine involvement is correlative, based on transcriptional signatures, rather than direct assays of thyroid hormone levels.

*The reviewer is correct that our inference of endocrine involvement is based on transcriptional signatures rather than direct measurements of thyroid hormone levels. Measuring TH concentrations in these juveniles is technically challenging because it requires sacrificing individuals and using whole-body extracts; the limited number of available juveniles prevented us from carrying out these assays in parallel with the present experiment. This is particularly sensitive in the treatments with anemones because if you put a too high number of juveniles there will be a lot of conflict between them and some will not enter in the sea anemone, therefore introducing an additional complication. Measuring TH level would necessitate a new series of replicates that is, as stated above currently impossible.*

*However, our previous work has demonstrated a strong correlation between transcriptomic signatures (including Tg, TPO, deiodinases, and TH receptor expression) and measured TH levels in anemonefish (see Roux et al., 2023, Cell Reports 42, 112661). This provides us with reasonable confidence that the observed transcriptional changes are a reliable proxy for underlying endocrine activity. Nevertheless, as requested by the reviewer, we have now added a cautionary statement to make explicit that this interpretation remains correlative and our interpretation is based on thyroid signaling activity inferred from the transcriptomic data (see Lines 701-703):*

*“****Although these transcriptional changes are consistent with shifts in thyroid hormone signaling, these findings remain correlative until TH levels are directly measured****.”*

*Overall, this study makes an important contribution by linking social context, cellular mechanisms, and evolutionary outcomes in a single system. By adding clarifying statements on sample sizes, species-specific scope, correlative framing, and the limits of gene-expression proxies for endocrine activity, the manuscript will present an appropriately cautious and balanced interpretation of the findings.*

*We hope that our above changes sufficiently address the Reviewer’s concerns and again thank them for their very kind appraisal.*

**Additional minor changes/corrections:**

- Line 138: Corrected “18” to “38”, indicating the correct age of fish.
- Lines 165-166: Added information on the third replicate to the Figure 1 legend.
- Lines 177-188: Replaced statistics with recalculated values which account for the addition of data from Replicate 3.
- Lines 199-200, 212-214: Updated statistics to incorporate body size data of fish from Replicate 3.
- Lines 240-241: Updated size ratio statistics to include Replicate 3 data.
- Line 648-649: Added artist permission statement for use of fish and anemone illustrations in Figure 7.
- Line 700: Added “also”.
- Lines 777-778: minor sentence revision to “Distinct ultraviolet/UV coloration can also signal subordinance in juveniles of the Barrier Reef anemonefish (*A. akindynos*) [32].”
- Lines 834-835: Added data availability statement for raw RNAseq dataset.
- Line 835-836: Replaced stated repository with a link to Zenodo hosted experimental dataset.
- Lines 838-851: Added missing acknowledgements for Marleen Klann, Patrick Mehlen, and Rachel C. Vella.
- Line 1114: Replaced “juveniles” with “fish” as the same method was used to measure both adult and juvenile standard length.
- Lines 1769-1840: Added Supplementary Figure legends (as requested).
- Figures 1D, 1E, S1: Updated plots to include Replicate 3 datapoints.
